# Comprehensive circular RNA profiling reveals the regulatory role of the circRNA-100338/miR-141-3p pathway in hepatitis B-related hepatocellular carcinoma

**DOI:** 10.1038/s41598-017-05432-8

**Published:** 2017-07-14

**Authors:** Xiu-Yan Huang, Zi-Li Huang, Yong-Hua Xu, Qi Zheng, Zi Chen, Wei Song, Jian Zhou, Zhao-You Tang, Xin-Yu Huang

**Affiliations:** 1 Department of General Surgery, Shanghai Jiaotong University Affiliated Sixth People’s Hospital, Shanghai, 200233 P.R. China; 2 Department of Radiology, Xuhui Central Hospital, Shanghai, 200031 P.R. China; 3 Thayer School of Engineering, Norris Cotton Cancer Center, Dartmouth College, Hanover, NH 03755 USA; 4 Howard Hughes Medical Institute; Department of Genetics, Harvard Medical School, Boston, MA 02115 USA; 5 Liver Cancer Institute and Zhongshan Hospital, Fudan University, Shanghai, 200032 P.R. China

## Abstract

Circular RNAs (circRNAs) represent a class of endogenous noncoding RNAs that have recently been recognized as important regulators of gene expression and pathological networks. However, their transcriptional activities and functional mechanisms in cancer remain largely unknown. Here, we present results from a global circRNA expression and functional analysis of patients with hepatocellular carcinoma (HCC). Using a circRNA microarray, we identified 226 differentially expressed circRNAs, of which 189 were significantly upregulated and 37 were downregulated. High expression of circRNA_100338, one of the upregulated circRNAs in HCC, is closely correlated with a low cumulative survival rate and metastatic progression in HCC patients with hepatitis B. Furthermore, our *in silico* and experimental analyses identified miR-141-3p as a direct target of circRNA_100338. Thus, circRNA_100338 functions as an endogenous sponge for miR-141-3p in HCC. In addition, we identified the crucial antagonistic roles of circRNA_100338 and miR-141-3p in the regulation of invasive potential in liver cancer cells. Overall, the differential expression of multiple circRNAs in HCC tissues and their clinical significance in hepatitis B-related HCC patients as revealed by our study suggests that circRNA_100338 is a potentially valuable biomarker for HCC diagnosis and target for HCC therapeutics.

## Introduction

Circular RNAs (circRNAs) represent a class of naturally occurring endogenous noncoding RNAs that have recently been recognized as important regulators of gene expression networks. The widespread presence of circRNAs with highly regulated temporal-specific or tissue-specific expression patterns has been identified in a variety of animals^1–3^. Evidence from computational analyses of expression data in multiple organisms suggests that circRNAs are created by RNA splicing events that occur at a characteristic “head to tail” splice junction, where an acceptor splice site at the 5′ end of an exon and a donor site at the 3′ end of a downstream exon are joined^1, 4^. In humans, most (~85%) circRNAs are transcribed from the sense strand of known protein coding genes, spanning across exons 1–5^1, 2^. Furthermore, sequences of circRNAs are conserved to some degree, suggesting their potential functions in biological processes^1, 4–6^.

In contrast to other linear RNAs, such as mRNAs or microRNAs (miRNAs), whose functions have been intensively studied in the past decades^7–10^, little is known about circRNAs, and even less is understood. Recent studies from two independent groups showed that one human circRNA derived from the antisense strand of the human Cerebellar Degeneration-Related protein 1 (CDR1) locus contains more than 70 endogenous miR-7 target recognition sites, thereby serving as a miR-7 sponge to “sponge up” or sequester the biological impacts of endogenous miR-7. This striking feature enables this circRNA, named CiRS-7 (Circular RNA Sponge for miR-7) or CDR1as (antisense), to function as a negative regulator of miRNA^1, 4^. Consistently, perturbation of CiRS-7 levels in both cell culture and neuronal tissues leads to an inverse change in endogenous miR-7 and dramatic changes in transcriptome profiles or developmental processes^1, 4^. Similarly, the testis-specific circRNA *Sry* functions as a miR-138 sponge^4^. These findings suggest that circRNAs play a crucial role in regulating gene expression and that alteration of circRNA expression may contribute to the pathogenesis of many diseases, including cancer.

In recent years, the impacts of ceRNA (competing endogenous RNA) interplay on the course of cancer initiation and progression have gradually emerged, and ceRNAs have been documented in various types of cancer, including prostate, liver and breast cancers^11, 12^. Given that circRNAs are potential ceRNAs, understanding circRNA transcriptional activities in cancer would greatly facilitate the study of cancer pathogenesis and provide potential novel targets for cancer therapeutics.

As one of the most malignant and common cancers worldwide, hepatocellular carcinoma (HCC) is the third leading cause of cancer mortality and has steadily spread from the eastern to western countries^13–15^. The development of HCC is a complex process that involves accumulation of gene regulation alteration at multiple levels, and molecules such as transcriptional factors, histone modifiers, microRNAs, lncRNAs and ceRNAs have been identified to play adominant role^16–20^. However, the exact roles of circRNAs in cancer and the underlying molecular mechanism of circRNA-mediated gene regulation during HCC development remain elusive.

The development of a circRNA microarray has greatly facilitated the understanding of circRNA expression in diverse biological contexts. In this study, we present a large-scale circRNA expression analysis of human HCC tissues. We found that multiple circRNAs exhibit differential expression in HCC tissues, suggesting their crucial roles in cancer development. The clinical significance of circRNA_100338 was also studied and its potential as a biomarker for HCC diagnostics is proposed. Computational analyses followed by experimental verification revealed that hsa_circRNA_100338 directly interacts with miR-141-3p in the context of HCC, thus sponging miR-141-3p for downstream gene regulation in HCC. In addition, we demonstrate the crucial antagonistic roles of circRNA_100338 and miR-141-3p in regulation of metastatic potential in liver cancer cells. Our data indicate that circRNAs potentially mediate gene expression in HCC, and provide one of the first circRNA biomarkers for HCC clinical studies.

## Results

### General profiles of circRNA in HCC

**Figure 1:**
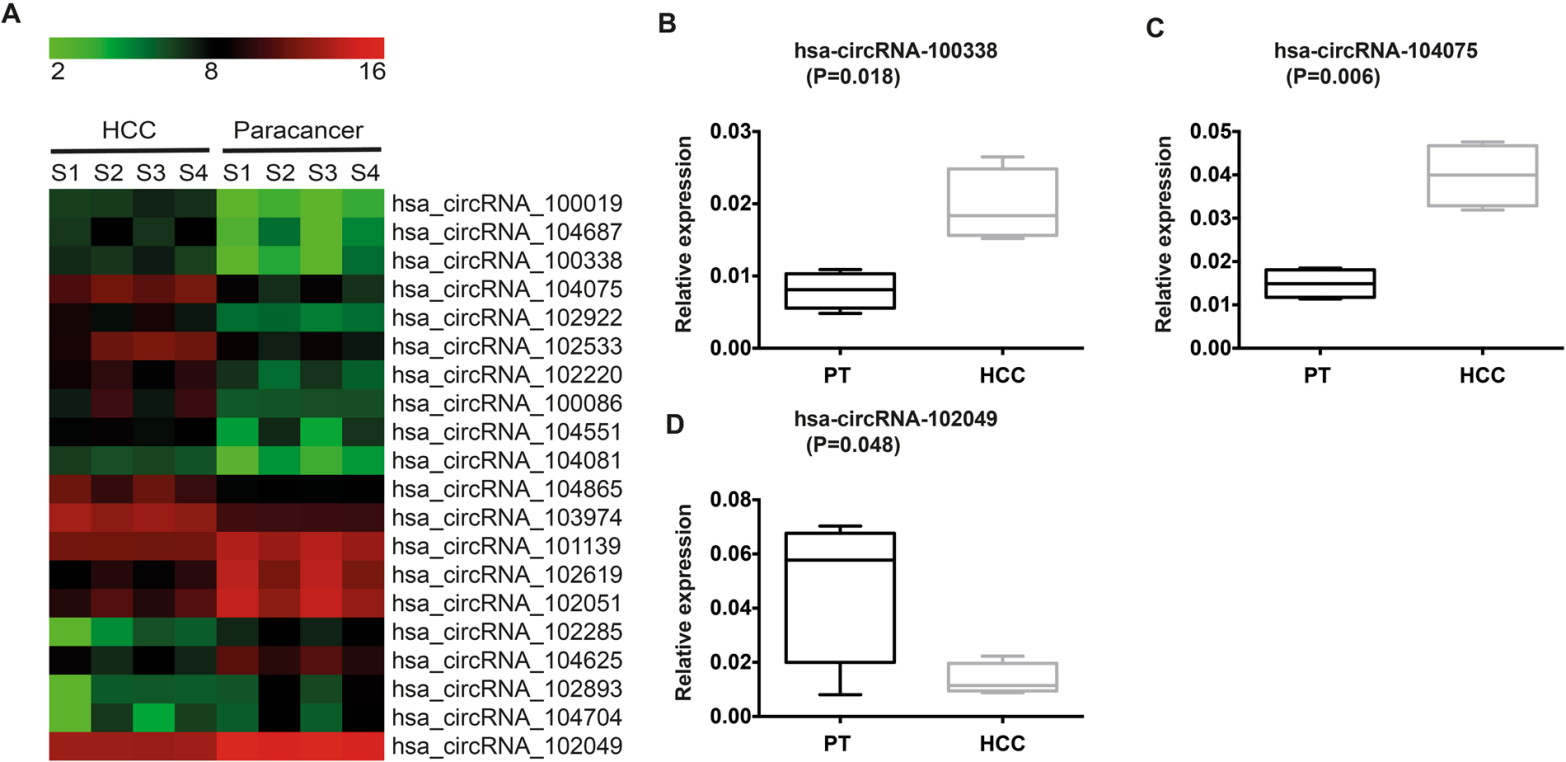
Differential expression of selected circRNAs in HCC and paired pericancerous tissues (PT) samples. (**A**) Based on the circRNA microarray results, the top 12 upregulated and top eight downregulated circRNAs in HCC compared with PT samples are shown in the heatmap. The red colour represents high expression, whereas the green colour represents low expression. (**B**–**D**) qRT-PCR verification of the expression of (**B**) circRNA_100338, (**C**) circRNA_104075 and (**D**) circRNA 102049 in HCC and paired PT samples (four patient samples; *n* = 4).

To identify circRNA-mediated regulation of gene expression profiles in human circRNA, we isolated RNAs from HCC tissues and paired pericancerous tissues from four patients (biological replicates) and performed circRNA microarrays to examine their expression profiles in each tissue. To increase the reliability of differential expression detected in the microarray between HCC and paired pericancerous tissues, circRNAs with low signal intensities or not expressed in all samples in the array were first filtered out (see Methods for details). Thus, among the remaining circRNAs, only the circRNAs that showed at least 2-fold expression change were considered (*P* < 0.05). Overall, analyses from the microarray resulted in the identification of a total of 226 differentially expressed circRNAs in HCC tissues, of which 189 were significantly upregulated and 37 were downregulated (Fig. 1A). To increase the accuracy and further narrow down the list, we evaluated all the differentially expressed circRNAs according to their overall raw signal intensities, statistical values and foldchange (FC), and selected six circRNAs for further study, of which four were upregulated (hsa_circRNA_104075, hsa_circRNA_100338, hsa_circRNA_102533 and hsa_circRNA_102922) and two were downregulated (hsa_circRNA_101139 and hsa_circRNA_102049) (Table 1).

**Table 1:** Top six circRNAs that show differential expression in HCC compared with paired pericancerous tissues in patients.

circRNAs	Fold-change	*P*-value	Host gene
hsa_circRNA_104075	11.1	0.021152	NUP153
hsa_circRNA_100338	11.5	0.037423	SNX27
hsa_circRNA_102533	7.3	0.047489	UBA2
hsa_circRNA_102922	7.6	0.009557	SGPP2
hsa_circRNA_101139	−2.9	0.007659	ATP2A2
hsa_circRNA_102049	−4.9	0.000257	TADA2A

### Experimental validation of circRNA expression using qPCR

**Figure 2:**
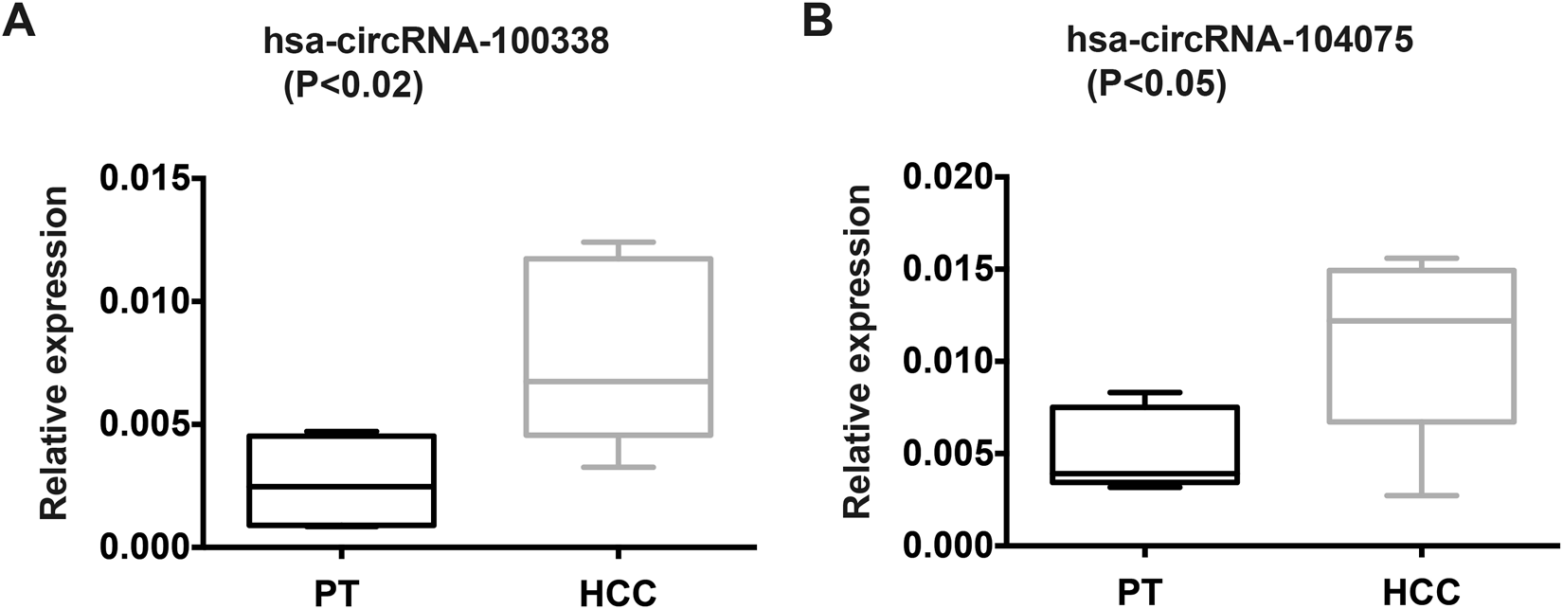
qRT-PCR verification of the expression of two circRNAs (**A**) circRNA_100338 and (**B**) circRNA_104075 in HCC and paired pericancerous tissues (PT) samples (additional six patient samples; *n* = 6).

To independently confirm the differential expression of these six circRNAs between HCC tissues and paired pericancerous tissues, we performed qRT-PCR to determine their expression levels in the HCC tissues and paired pericancerous tissues used for the above microarray analyses. Among the six circRNAs, expression of four of the circRNAs was detected in HCC tissues and pericancerous tissues, and three of the four showed differential expression patterns similar to those identified in our microarray analyses, as shown in Fig. 1B–D, suggesting the validity of our approach and the potential gene regulatory roles of these three circRNAs in HCC. The remaining two circRNAs were not detected in our qPCR experiments, presumably due to expression levels below the limit of detection. We also verified the differential expression of hsa_circRNA_102533 and found that its expression difference between HCC and paired pericancerous tissues was not significant (*P* = 0.2638). Therefore, we removed this circRNA from further investigation in this study. To further confirm that the remaining three circRNAs are subjected to specific regulation in HCC, we performed qRT-PCR to determine their expression levels in HCC and paired pericancerous tissue samples derived from an additional six HCC patients (Fig. 2). Despite the great genomic variations among the different patients, we were still able to identify two circRNAs (hsa_circRNA_104075 and hsa_circRNA_100338) that were significantly up-regulated in HCC samples compared with paired pericancerous tissue samples. These results further validated our findings and implied that these circRNAs may play important roles in HCC carcinogenesis.

### Clinical impact of circRNAs in HCC patients

Ectopic expression of certain circRNAs in HCC tissues but not in paired pericancerous liver tissue samples raised an important question of whether the expression levels of these highly expressed circRNAs are clinically correlated with the development of HCC. We collected HCC and paired pericancerous tissue samples from an additional 80 clinical patients with hepatitis B and explored the possible connections between circRNA expression and several clinicopathological measurements in patients. For simplicity, we selected circRNA_100338 as a model for downstream analyses, mainly due to its relatively large differential expression in HCC tissues.

**Figure 3:**
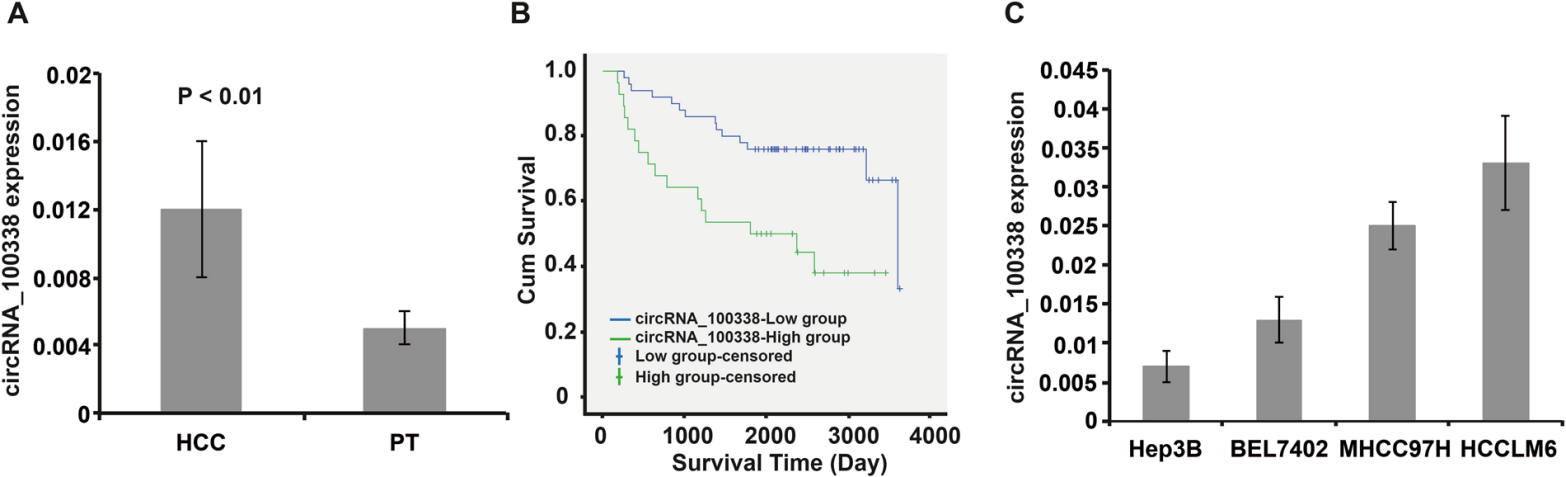
Expression level of circRNA_100338 is correlated with patient cumulative survival rate and HCC metastasis. (**A**) qRT-PCR verification of circRNA_100338 expression in HCC tissues and paired pericancerous tissues (PT) samples (total of 80 patient samples; *n* = 80). (**B**) Longer survival in the circRNA_100338-low group (low group) compared with the circRNA_100338-high group (high group) was observed; *P* < 0.01. In this study, “censored” refers to survival cases at the end of follow-up. (**C**) HCC cell lines with higher metastatic potential expressed a higher level of circRNA_100338. Statistical testing was performed with one way ANOVA; *P* < 0.001.

We first assessed circRNA expression in these 80 HCC patients. As expected, a higher level of circRNA_100338 was observed in HCC tissues compared with pericancerous liver tissues (*P* < 0.01) (Fig. 3A). Within 80 HCC tissues, circRNA_100338 displayed diverse expression levels despite high expression relative to pericancerous tissues; we thus further divided HCC patients into two groups according to their circRNA_100338 expression levels in HCC tissues (see Methods): circRNA_100338-low group (circRNA_100338/GAPDH ≤ 0.015, *n* = 51) and circRNA_100338-high group (circRNA_100338/GAPDH > 0.015, *n* = 29) and evaluated the patient cumulative survival rate in these two groups. Survival and metastasis data for two of the patients were missing. The overall survival rate was 61.5% within the remaining 78 cases. Strikingly, the cumulative survival rate (72.0%) of HCC patients in the circRNA_100338-low group was significantly higher than that (42.9%) of HCC patients in the circRNA_100338-high group (*P* < 0.02), suggesting that circRNA_100338 may serve as a marker for malignancy diagnosis in HCC. The survival of the circRNA_100338-low group (low group) was significantly longer than that of the circRNA_100338-high group (high group) (Fig. 3B), but the curves did not meet based on our current data. However, one case in the low group died at day 3603, which led to the abrupt downturn of the survivorship curve of the low group. If we extended the follow-up period, the curve of the high group would also go down abruptly. To identify the possible underlying mechanisms, we further collected several related physiological and pathological data from these 80 patients and analysed the correlations between this dataset and the circRNA_100338 expression levels. Corresponding with the results described above, these results indicated that the expression levels of circRNA_100338 were not significantly related to the age or gender of the HCC patients in our sample but were significantly correlated with many metastatic clinicopathological parameters, such as TNM stage, vascular invasion and lung metastasis (Table 2), indicating that circRNA_100338 affects the cumulative survival rate of HCC patients through at least regulation of metastasis. In agreement with these findings, examination of HCC cell lines (*in vitro*) with increased metastatic potential (Hep3B, BEL7402, MHCC97H and HCCLM6) revealed progressively increased expression of circRNA_100338 (Fig. 3C), consistent with the identified correlation of circRNA_100338 with cumulative survival rates and metastasis in HCC patients. Together, our data suggested that expression levels of circRNA_100338 in HCC patients are highly correlated with cancer metastatic progression and, consequently, the cumulative survival rate. Thus, circRNA_100338 may be a potential biomarker for clinical diagnosis and evaluation.

**Table 2:** Correlations between tumour circRNA_100338 expression level and clinicopathological parameters of HCC patients.

Clinicopathological parameters	*n*	circRNA_100338/GAPDH (*n*)		*P*
		Low group	High group	
		(≤0.015, *n* = 51)	(>0.015, *n* = 29)	
Age	80			0.847
≤55 y	43	27	16	
>55 y	37	24	13	
Sex	80			0.411
Female	18	10	8	
Male	62	41	21	
Cirrhosis	80			0.794
Yes	65	41	24	
No	15	10	5	
TNM stage^**^	80			0.032^*^
I–II	43	32	11	
III–IV_A_	37	19	18	
Vascular invasion	80			0.012^*^
Yes	49	26	23	
No	31	25	6	
Lung metastasis	78^#^			0.002^†^
Yes	12	3	9	
No	66	47	19	

NOTE: HCC, hepatocellular carcinoma; ^#^Number less than 80 due to missing data. Significant difference: *P < 0.05, ^†^ *P* < 0.01. ^**^The TNM classification of malignant tumours (TNM) is a cancer staging system that describes the stage of a cancer that originates from a solid tumour with alphanumeric codes, using the size and extension of the primary tumour, its lymphatic involvement, and the presence of metastases to classify the progression of cancer. T: size or direct extent of the primary tumour; N: degree of spread to regional lymph nodes; M: presence of distant metastasis.

### circRNA_100338 interacts with miR-141-3p

**Figure 4:**
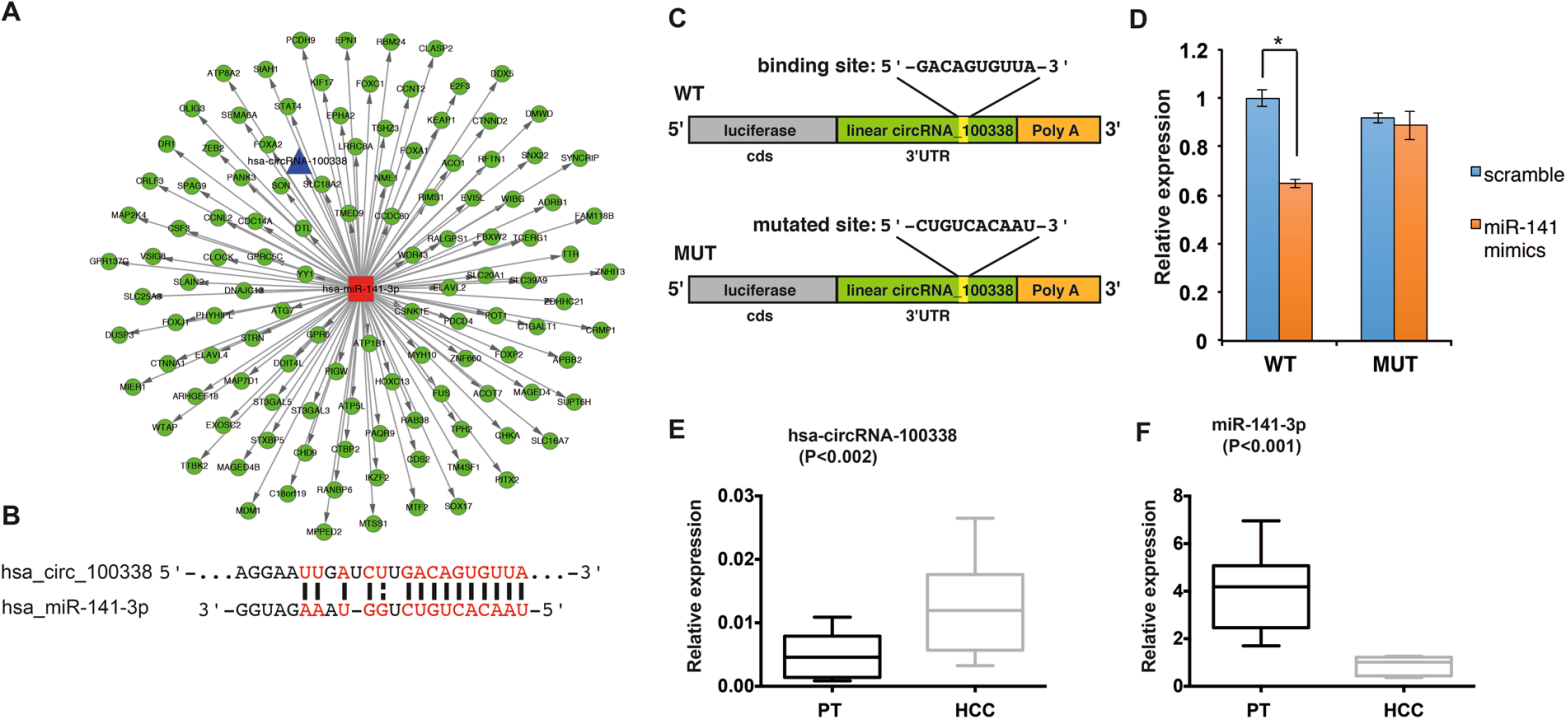
circRNA_100338 interacts with miR-141 in HCC. (**A**) A biomathematically predicted miRNA-target network for hsa-miR-141-3p. This network is predicted based on sequence-pairing prediction. miRNA and its predicted target genes are labelled in red and green, respectively. Specifically, circRNA-100338 is also indicated as a target (presented in blue). (**B**) Sequence base-paring between circRNA_100338 and miR-141-3p. Note that the seed region is fully complementary to the sequence of circRNA_100338. (**C**) Schematic of luciferase reporter genes. Linearized circRNA_100338 sequence with a wild-type (WT) or mutated (MUT) miR-141-3p interaction site was fused into the 3′ UTR of the luciferase gene. (**D**) The luciferase reporter assay indicated that circRNA_100338 directly interacts with miR-141-3p at the predicted interaction site in a human cellular context. Assays were performed using either miR-141 mimic (orange) or scramble RNA control (blue). (**E**) Further verification of circRNA_100338 expression in HCC and paired pericancerous tissues (PT) using qRT-PCR (*n* = 10). (**F**) Verification of miR-141-3p expression in HCC and paired pericancerous tissues (PT) samples (*n* = 10). *n* = 3; *P = 0.003.

Despite the broad expression of circRNAs in diverse cells and tissues, their cellular and biological functions remain largely elusive. Given the cases where circRNAs “sponge up” miRNAs to promote expression of miRNA target genes, we speculated that these two upregulated circRNAs likely regulate gene expressions by interacting with endogenous miRNAs^1, 4^. Therefore, we performed *in silico* analyses to predict miRNAs targeted by these two circRNAs. For each of the circRNAs, many miRNAs have been predicted as potential targets (Fig. 4A) as expected. When compared with the expression in pericancerous tissue, miR-141-3p expression in HCC decreased abruptly compared with miR-200a-3p expression. To further narrow down the target list and remove the false-positives, we focused only on miRNAs that are highly expressed in cancer cells. Among all the miRNAs identified, miR-141-3p is particularly worth investigating, due to the following reasons: (1) miR-141-3p binding sites of high complementarity were found in hsa_circRNA_100338 with a high prediction score, suggesting a strong likelihood of miR-141-3p being a bona fide circRNA target (Fig. 4B); (2) miR-141-3p is widely dysregulated in several cancer types, such as colorectal cancer, bladder cancer, ovarian cancer and liver cancer^21–25^; and (3) miR-141-3p has previously been implicated as a tumour or metastasis suppressor in various types of cancer cells^24, 26^. These lines of evidence suggest that circRNA_100338 may interact with miR-141-3p to regulate the gene expression necessary for HCC carcinogenesis.

We next sought to test whether or not circRNA_100338 is capable of sponging miR-141-3p in a cellular context. To validate our hypothesis, we fused the linearized sequence of circRNA_100338 (with a wild-type (WT) or mutant miR-141-3p binding site) into the 3′ UTR of the reporter gene Renilla luciferase and performed a Dual-Luciferase Reporter Assay in human embryonic kidney 293T (HEK293T) cells (Fig. 4C). As expected, luciferase expression significantly decreased when HEK293T cells were co-transfected with miR-141 mimic, compared with the control cells co-transfected with scramble RNAs. Consistently, co-transfection with miR-141-3p mimic or co-transfection with scramble RNA in circRNA_100338-expressing HEK293T cells led to comparable expression of luciferase when the miR-141-3p binding site was mutated in circRNA_100338 (Fig. 4D). These data indicated a highly efficient interaction between circRNA_100338 and miR-141-3p *via* the predicted binding site in a human cellular context and provided direct evidence of sponging of miR-141-3p by circRNA_100338 *in vivo*.

To confirm the interaction between circRNA_100338 and miR-141-3p, we further analysed the expression of miR-141-3p using qRT-PCR in the ten HCC tissue and paired pericancerous tissue samples that we previously used for hsa_circ_100338 expression analysis. As expected, the expression of circRNA_100338 was significantly upregulated by approximately 3~4-fold in HCC tissues compared with that in paired pericancerous tissues (Fig. 4E). In sharp contrast to circRNA_100338, the expression of miR-141-3p in HCC tissues was downregulated compared with that in paired pericancerous tissues by approximately five fold and with high statistical significance (*P* < 0.001) (Fig. 4F). Taken together, our data strongly suggest that hsa_circRNA_100338 functions, at least in part, by directly interacting with miR-141-3p in HCC.

### miR-141-3p antagonizes circRNA_100338 to regulate HCC metastatic potential

**Figure 5:**
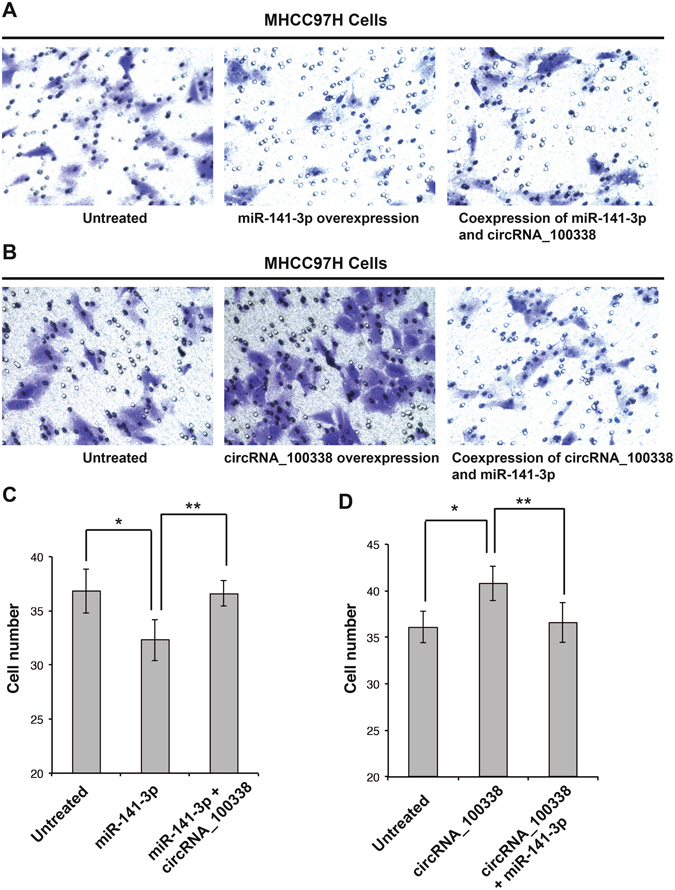
circRNA_100338 and miR-141-3p regulate the metastatic potential of liver cancer cells. (**A**) *In vitro* invasion assay in MHCC97H cells overexpressing miR-141-3p or co-expressing both miR-141-3p and circRNA_100338. (**B**) *In vitro* invasion assay in MHCC97H cells overexpressing circRNA_100338 or co-expressing both circRNA_100338 and miR-141-3p. (**C**) The number of invasive cells in assays of MHCC97H cells overexpressing miR-141-3p. Data are presented as the means ± SE of three assays. *n* = 3; *P = 0.013; **P = 0.004. (**D**) The number of invasive cells in assays with MHCC97H cells overexpressing circRNA_100338. Data are presented as the means ± SE of three assays. *n* = 3; *P = 0.005; **P = 0.019.

Given that circRNA_100338 interacts with miR-141-3p and that circRNA_100338 is positively correlated with metastasis in HCC patients, we next determined whether miR-141-3p can counteract circRNA_100338 and inhibit cell metastatic progression in HCC. We first overexpressed miR-141-3p in the liver cancer cell line MHCC97H, and then performed *in vitro* invasion assays to test the metastatic potential of miR-141-3p-overexpressing MHCC97H cells. The principle of this assay is based on two medium-containing chambers separated by a porous membrane through which cells can transmigrate. Generally, cells seeded in medium in the upper chamber migrate vertically through the membrane pores into the lower compartment, in which medium containing an attractant or simply a higher serum level is present. The migratory and invasive capacities of tumour cells are determined by the number of cells that invade the membrane after 24 h of incubation. Invasive cells were fixed and stained with cytological dyes for counting. As indicated in Fig. 5A, overexpression of miR-141-3p in MHCC97H cells resulted in a much smaller number of penetrated cells (purple cells) compared with the untreated control. This downregulated invasive capacity was restored when the cells co-expressed both miR-141-3p and circRNA_100338. In line with the above findings, when circRNA_100338 was overexpressed, MHCC97H cells displayed enhanced migratory and invasive ability, and many more invasive cells were found compared with the untreated control (Fig. 5B). As expected, the enhanced invasive capacity induced by circRNA_100338 could be rescued by elevated expression of miR-141-3p. We counted the cells in each treatment and found that miR-141-3p-expressing cells decreased the metastatic potential by 12.1% and that the metastatic potential was restored when circRNA_100338 was co-expressed (Fig. 5C). Accordingly, overexpression of circRNA_100338 led to an increase of 12.9% in the metastatic potential compared with the untreated control (Fig. 5D). Together, our data strongly indicated that circRNA_100338 positively regulates metastatic potential in liver cancer cells, whereas miR-141-3p functions as an antagonist of circRNA_100338 to inhibit cancer cell metastasis. This result is consistent with the identified expression pattern of circRNA_100338 and miR-141-3p in HCC patients and provides direct evidence of circRNA involvement in regulation of metastatic progression in liver cancers.

## Discussion

As a novel gene regulator, circRNAs are potentially involved in multiple biological and pathological processes^1, 4, 27–31^. However, very little is known regarding their roles in cancer. In this study, we investigated the circRNA expression profile in HCC and paired pericancerous tissues and found that 189 of 226 differentially expressed circRNAs were significantly upregulated and 37 were downregulated in HCC. Specifically, we showed that circRNA_100338 is upregulated in HCC compared with paired pericancerous tissues, which significantly affects the cumulative survival rate and cancer metastasis in HCC patients. The follow-up period in this study was at least 5 years; thus, we think we can derive a firm conclusion even though the sample number is relatively low. In addition, our study indicated that circRNA_100338 sequesters miR-141-3p in the context of HCC tissue. Despite the small number of circRNA reports in HCC studies, to our knowledge, this study is one of the first few differential expression analyses of circRNAs reported for HCC. More importantly, we identified a novel circRNA biomarker for HCC clinical diagnosis and patient survival estimates.

Since the first report of circRNA functioning as a miRNA sponge, the potential of circRNAs in regulating cancer-related genes through fine-tuning miRNAs has recently been recognized. To date, it has been well established that miR-7 directly targets many oncogenic factors and is involved into multiple cancer-related signalling pathways, including EGFR, IRS-1/2, Raf1, Pak1, Ack1, PA28-gamma, IGF1R, PIK3CD and mTOR^32–38^. Therefore, as a circular miR-7 inhibitor, the newly identified CiRS-7 potentially plays an important role as a putative oncogene in cancer. However, knowledge regarding a direct connection between circRNAs and cancer is still very limited. Currently, the correlation between individual circRNAs and cancer has been explored for very few cancer types, such as colorectal cancer, ovarian cancer, bladder cancer and gastric cancer^39–41^. Nonetheless, the downstream interacting miRNAs as well as their regulated protein coding genes in those cancers are still missing, although individual circRNAs, such as hsa_circ_002059 have been reported to be significantly downregulated in gastric cancer. Recently, two circRNA studies also indicated that hsa_circ_0005075 and hsa_circ_0001649 are differentially regulated in HCC tissues^42, 43^. In our study, we further expanded the scope of exploration in HCC and performed a more comprehensive, large scale circRNA expression analysis. We identified at least two differentially expressed circRNAs (hsa_circRNA_104075 and hsa_circRNA_100338) in HCC. Importantly, our clinical evidence from 80 HCC patients further indicated that ectopic expression of circRNA_100338 was always accompanied by a decreased cumulative survival rate, elevated vascular invasion and lung metastasis in HCC patients, providing a potential circRNA biomarker for HCC diagnosis and patient survival rate estimation. Moreover, our study identified miR-141-3p as part of the underlying mechanism of hsa_circRNA_100338-mediated HCC carcinogenesis, presenting the first functional model of a circRNA during carcinogenesis in the liver. Given that the interaction between circRNA and miRNA may not be exclusive, these two upregulated HCC-associated circRNAs may target other cancer-associated miRNAs in HCC tissues. Alternatively, because each miRNA may have multiple mRNA targets, hsa_circRNA_100338 is also likely to target more oncogenes by interacting with miR-141-3p.

With the discovery of CiRS-7 and *Sry*, circRNAs have shown huge potential for interaction with endogenous miRNAs. In our study, we showed that hsa_circ_100338 is upregulated in HCC tissues and can target miR-141-3p; thus, hsa_circ_100338 may serve as an important gene regulator in HCC tissues. Currently, many miRNAs are associated with cancer-related signalling pathways; by contrast, studies of the circRNA-miRNA-mRNA network are still lacking. Using computational analyses followed by experimental verification, we provide the first identification of a miRNA that potentially directly interacts with circRNA_100338. Furthermore, *in vitro* invasion assays in MHCC97H cells, a metastatic liver cancer cell line, provided direct evidence of the involvement of circRNA_100338 and miR-141-3p in regulation of metastasis in liver cancers. Given than each miRNA targets multiple downstream genes, it would be very interesting to identify the downstream miR-141-3p target genes that are responsible for the regulation of cancer cell metastasis. In fact, our *in silico* analyses of miR-141-3p target genes have revealed that metastasis suppressor 1 (*MTSS1*) is very likely a potential target of miR-141-3p, as indicated by highly conserved miRNA recognition elements (MREs) at the 3′ UTR of *MTSS1*. Even though *MTSS1* is widely known as a metastasis suppressor gene that is involved in regulation of cell mobility and consequently cancer metastasis^44–49^, recent studies surprisingly indicated that *MTSS1* also acts as an oncogene and a driver of metastasis in melanoma tumours and breast cancers^50, 51^. This evidence indicates that *MTSS1* may also serve as a metastasis driver in HCC patients and that circRNA_100338 regulates HCC metastasis though a potential circRNA_100338-miR141-3p-MTSS1 interaction pathway. Indeed, qRT-PCR performed in HCC and paired pericancerous tissues in this study also indicated that the expression of *MTSS1* is significantly upregulated in HCC tissues compared with paired pericancerous tissues, an inverse expression pattern to that of miR-141-3p. Therefore, it is very likely that miR-141-3p can function as a novel tumour suppressor in HCC. This speculation is also specifically supported by serum miRNA analysis in hepatitis B virus-related HCC^52^.

## Conclusion

Using a circRNA microarray, we determined that out of a total of 226 differentially expressed circRNAs in HCC tissues, 189 were significantly upregulated and 37 were downregulated. Specifically, circRNA_100338 is upregulated in HCC compared with paired pericancerous tissues and highly correlated with the cumulative survival rate and cancer metastasis in HCC patients. In addition, our study identified miR-141-3p as a direct downstream target of circRNA_100338 in the context of HCC tissue, and functions antagonistically with circRNA_100338 to regulate cell metastasis in liver cancers. To our knowledge, this study is one of the first two global circRNA differential expression analyses in HCC. In addition, our study provided a novel circRNA biomarker for hepatitis B-related HCC clinical diagnosis and patient survival estimation.

## Methods

### Clinical specimens

Four pairs of snap-frozen HCC tissue and matched para-carcinoma tissue were obtained from the Hospital Clinic for circRNA microarray analysis. Subsequently, a total of tenpaired samples, including the samples for microarray analysis, were used for circRNA validation using reverse transcriptase quantitative (RT-q) PCR. All the experimental subjects were consecutive patients and did not receive any other treatment prior to operation. All HCC cases were confirmed by experienced pathologists. Four T2 stage HCC samples were used for the circRNA microarray assay, and then six T1–T4 stage HCC samples were applied for circRNA validation using qPCR. Clinical and pathological characteristics of patients were determined according to WHO/ISUP classification and UICC TNM classification (2010) and are presented in Table 3.

**Table 3:** Clinical pathological characteristics of study subjects.

For circRNA microarray assay					For circRNA validation by qPCR		
Subject	Age	Gender	Stage	Grade	Samples		*n* = 10
1	56	Male	T2	G2	Median age, y (range)		57, (47~69)
2	49	Male	T2	G2	Gender	Male	6
3	67	Female	T2	G2		Female	4
4	51	Female	T2	G2	TNM Stage	T1	1
						T2	6
						T3	2
						T4	1
						N0	8
						N1	2
						M0	10
						M1	0
					Grade	G1	2
						G2	5
						G3	2
						G4	1

### HCC and pericancerous tissue separation

Between January 2006 and December 2010, 80 HCC patients underwent open hepatectomy by the same surgical team in our centre. All specimens were collected in the operating room immediately (≤15 min) after tissue removal and were snap frozen in liquid nitrogen and stored at −80 °C. For the 80 para-carcinoma controls, tissues adjacent to carcinoma, which were diagnosed as normal tissue using pathological methods, were taken from tissue ≥2 cm away from the tumour in HCC patients. HCC and pericancerous tissues were differentiated via haematoxylin and eosin (H&E) staining.

### circRNA microarray hybridization

Total RNA from either HCC or paired paracancerous tissues from each patient was extracted using TRIzol and quantified using a NanoDrop ND-1000. An aliquot of the RNA from each sample was reserved for downstream qRT-PCR analysis. Sample labelling and array hybridization were performed according to the manufacturer’s protocol (Arraystar Inc.). Briefly, circRNA was treated with Rnase R (Epicentre, Inc.) to remove linear RNAs. Then, each sample was amplified and transcribed into fluorescent cRNA utilizing a random priming method (Arraystar Super RNA Labeling Kit; Arraystar). The labelled cRNAs were purified using an RNeasy Mini Kit (Qiagen). The concentration and specific activity of the labelled cRNAs (pmol Cy3/μg cRNA) were measured using the NanoDrop ND-1000. One μg of each labelled cRNA was fragmented by adding 5 μl of 10× Blocking Agent and 1 μl of 25× Fragmentation Buffer. Then, the mixture was heated 60 °C for 30 min, and finally, 25 μl of 2× Hybridization buffer was added to dilute the labelled cRNA. Subsequently, 50 μl of hybridization solution was dispensed into the gasket slide and assembled onto the circRNA expression microarray slide. The slides were incubated for 17 h at 65 °C in an Agilent Hybridization Oven. The hybridized arrays were washed, fixed and scanned using an Axon GenePix 4000B microarray scanner (Molecular Devices, Inc.).

### circRNA microarray analysis

Scanned images were imported into GenePix Pro 6.0 software (Axon) for grid alignment and raw data extraction. Quantile normalization of raw data and subsequent data processing were performed using the R software package. After quantile normalization of the raw data, low intensity filtering was performed, and the circRNAs associated with at least four of eight samples with an “expressed” flag (greater than two times background standard deviation) were retained for further analyses. Then, by comparing two groups of profile differences, the “FC” between the groups for each circRNA was computed. Only the circRNAs that exhibited FCs greater than 2.0 and Student’s *t*-test *P*-values of less than 0.05 were identified as differentially expressed circRNAs. All experiments were performed and analysed in triplicate.

### Validation of candidate circRNAs using qRT-PCR

Total RNA extracted from cells of each patient was reverse transcribed using random primers, and quantitative PCR assays of cDNA were performed using a CFX96 Real-time PCR system (Bio-Rad) to evaluate the abundance of target transcripts relative to the house-keeping genes U6 or GAPDH^53^. Specifically, expression values in HCC and paired pericancerous tissues from each patient were first assessed by qRT-PCR independently. A histogram or box and whisker plots were then generated based on the values from the independent measurements of all patients. Target cDNAs were amplified using the following probe set:

GAPDH_F: 5′-GGGAAACTGTGGCGTGAT-3′

GAPDH_R: 5′-GAGTGGGTGTCGCTGTTGA-3′

hsa_circRNA_100338_F: 5′-AAAAGCAAGCAGTGCCCATA-3′

hsa_circRNA_100338_R: 5′-GCTCGAATCAGGTCCACCA-3′

hsa_circRNA_102922_F: 5′-GCCTTCACCCTCCTTATCTCTA-3′

hsa_circRNA_102922_R: 5′-TGGCATTCCATATTCAGCGA-3′

hsa_circRNA_104075_F: 5′-GAAGATGTCAAGCCCTTTAGC3′

hsa_circRNA_104075_R: 5′GAGTTGCTTAGCTTTCATTTGTC-3′

hsa_circRNA_101139_F: 5′-CATCCGCTACCTCATCTCGT-3′

hsa_circRNA_101139_R: 5′-GTTGCTACCACCACTCCCATA-3′

hsa_circRNA_102049_F: 5′-GAAGCATTTCATCAATAACCCTC-3′

hsa_circRNA_102049_R: 5′ –CAAAGCCACAGTCCATCACAG-3′

hsa_circRNA_102533_F: 5′-GCTGCCAAAAGCATAACCAA-3′

hsa_circRNA_102533_R: 5′-CCCCTTTTCTGCTAAATGAACTCT-3′

### *In vitro* invasion assay

The untreated control or treated MHCC97H cells (5 × 10^4^ cells/well) were added to the upper chamber (in 100 μl of DMEM), and 600 μl of conditioned medium was added to the lower chamber; chambers were separated by a porous membrane. The pore size of the membranes was determined by the size of the cells. After 24 h of incubation, the cells in the lower chamber were fixed with methanol and stained with crystal violet solution. The results were expressed as the number of penetrated cells as assessed using a microscope at 200x magnification and analysing five random fields. Results are presented as the means ± SD of three assays.

The miR-141-3p mimic and miR-141-3p inhibitor were synthesized by Ribobio (Guangzhou, China) and their respective sequences are as follows:

miR-141-3p inhibitor: 5′-CCAUCUUUACCAGACAGUGUUA-3′

miR-141-3p mimics: 5′-UAACACUGUCUGGUAAAGAUGG-3′

### Luciferase activity assays

Cells were co-transfected with circRNA-100338 plasmids or their mutant fragments andmiR-141-3P mimic by using Lipofectamine 2000 (Invitrogen, Foster city, CA) according to the manufacturer’s protocol. Firefly and Renilla luciferase activities were measured consecutively using a Dual-Luciferase Reporter Assay System (Promega, Massachusetts, USA) after transfection for 48 h. Each assay was repeated in six independent experiments.

### circRNA overexpression

The following is the sequence of circRNA_100338: GAACCACGUGAAUGUUGAGGGGGCGACACACAAGCAGGUGGUGGACCUGAUUCGAGCAGGCGAGAAGGAAUUGAUCUUGACAGUGUUAUCUGUACCUCCUCAUGAGGCAGAUAACCUAGAUCCCAGUGACGACUCGUUGGGACAAUCAUUUUAUGAUUACACAGAAAAGCAAGCAGUGCCCAUAUCGGUCCCCAGAUACAAACAUGUGGAGCAGAAUGGUGAGAAGUUUGUG. To transcribe the circRNA-100338 transcript, a circRNA-100338 overexpression vector was constructed. The specially designed front and back circular frames were synthesized and added to pCDH-CMV-MCS-EF1-copGFP for circulation of the transcripts. The front circular frame contains the endogenous flanking genomic sequence with an EcoRI restriction enzyme site, and the back circular frame contains part of the inverted upstream sequence with a BamHI site. The cDNA encoding circTCF25 in HeLa cells was amplified using the following primers:

5′-cgGAATTCTGAAATATGCTATCTTACAGGAACCACGTGAATGTTGAGG-3′

5′-cgGGATCCTCAAGAAAAAATATATTCACCACAAACTTCTCACCATTCTG-3′

As a result, the 232 bp target fragment (in order) contains an EcoRI site, splice acceptor AG, the circRNA-100338 sequence, splice donor GT and a BamHI site. Then, the amplified fragment was cloned into the vector between the two frames. In addition, we also established a mock vector containing a nonsense stuffer between the two circular frames rather than the circRNA-0000130-encoding cDNA. The vector construction was verified by direct sequencing. The vectors were constructed with help from Guangzhou Geneseed Biotech Co, Guangzhou, China.

### Patients, specimens and follow-up

Between January 2006 and December 2010, 80 HCC patients who underwent open hepatectomy by the same surgical team in our centre were recruited based on the diagnosis of HCC. These HCC patients were also infected with hepatitis B virus. The inclusion criteria for patients in this study were as follows: (a) patients with hepatitis B from 2006 to 2010; (b) no anticancer treatment prior to hepatectomy; (c) pathologically proven HCC based on WHO criteria; (d) availability of frozen resected HCC tissues and follow-up data. This study was approved by the Research Ethics Committee of Shanghai Jiaotong University affiliated Sixth People’s Hospital, and informed consent was obtained from each patient. All methods were performed in accordance with the relevant guidelines and regulations. The determination of HCC patient grouping was derived from the average value of hsa_circRNA_100338/GAPDH from all HCC patients (0.005), and the cutoff of the circRNA_100338-high group was set as 0.015, which is 3 times more than average value. Patients with hepatectomy were followed up with every two months during the first postoperative year and at least every four months subsequently until December 2015 via monitoring abdominal ultrasonography, chest X-ray or computed tomography depending on the patient’s condition. General data, metastatic characteristics, pathological characteristics and survival were compared between the two groups. The cumulative survival rate presented in Fig. 3B was generated using the records up to Dec. 31, 2015. Of the 29 patients in the circRNA_100338-high group, one was lost to follow-up and 16 died in cumulation; of the 51 patients in the circRNA_100338-low group, one was lost and 14 died in cumulation.

### miRNA target prediction

Target genes of hsa-miR-141-3p were predicted independently using three different algorithms: miRanda (http://www.microrna.org/microrna/home.do)^54^, TargetScan (Version 6.0, http://www.targetscan.org/vert_60/)^55^ and MicroCosm (Version 5, http://www.ebi.ac.uk/enright-srv/microcosm/htdocs/targets/v5/). Only the genes that overlapped in the results of at least two algorithm predictions were considered.

### Statistical analysis

Quantile normalization and subsequent data processing were performed using the R software package. All other statistical data were analysed and visualized with GraphPad Prism 6.0 software (GraphPad Software, La Jolla, CA). The qPCR validation of all samples tested by a paired *t*-test, and *P* < 0.05 was considered statistically significant. All continuous variables were expressed as the means ± SD or means ± SE. RNA or cells from one patient were considered as one sample. qRT-PCR measurement of each sample was performed and analysed in triplicate. The normality of the distribution of values in each group was verified using SPSS software. ANOVAs was used for statistical comparison among groups. *hsa_circRNA_100338* expression and clinicopathological parameters were analysed using a Pearson chi-square test or Fisher’s exact test. The survival analysis was conducted using the Kaplan-Meier method (log-rank test).

### Ethics statement

This study was approved by the Research Ethics Committee of Shanghai Jiaotong University affiliated Sixth People’s Hospital, and informed consent was obtained from each patient. All specimens were collected in the operating room immediately (≤15 min) after tissue removal and were snap frozen in liquid nitrogen and stored at −80 °C. All methods were performed in accordance with the relevant guidelines and regulations.
